# Evaluation of the Antimicrobial Efficacy of Sodium Hypochlorite, Ozone, Diode Laser, and Photodynamic Therapy Against *Enterococcus faecalis* and *Streptococcus mutans*: An In-Vitro Study

**DOI:** 10.3290/j.ohpd.c_2454

**Published:** 2026-01-20

**Authors:** Cagdas Ozkan, Sera Simsek Derelioglu, Hayrunisa Hanci, Nazli Nur Aslan Ince, Fatih Sengul, Elif Buse Elif Kaplan, Peris Celikel

**Affiliations:** a Cagdas Ozkan Research Assistant, Ataturk University Faculty of Dentistry, Department of Pediatric Dentistry, Erzurum, Turkey. Methodology, data curation, reviewed and edited manuscript, contributed substantially to the conception and design of the study.; b Sera Simsek Derelioglu Professor, Ataturk University Faculty of Dentistry, Department of Pediatric Dentistry, Erzurum, Turkey. reviewed and edited manuscript, contributed substantially to the conception and design of the study.; c Hayrunisa Hanci Associate Professor, Ataturk University Faculty of Pharmacy, Erzurum, Turkey. Methodology, formal analysis, reviewed and edited manuscript, contributed substantially to the conception and design of the study.; d Nazli Nur Aslan Ince Research Assistant, Ataturk University Faculty of Dentistry, Department of Pediatric Dentistry, Erzurum, Turkey. Investigation, visualization, contributed substantially to the conception and design of the study.; e Fatih Sengul Associate Professor, Ataturk University Faculty of Dentistry, Department of Pediatric Dentistry, Erzurum, Turkey. Methodology, formal analysis, wrote original draft, reviewed and edited manuscript, contributed substantially to the conception and design of the study.; f Elif Buse Elif Kaplan Research Assistant, Ondokuz Mayıs University Faculty of Dentistry, Department of Pediatric Dentistry, Samsun, Turkey. Investigation, data curation, contributed substantially to the conception and design of the study.; g Peris Celikel Assistant Professor, Ataturk University Faculty of Dentistry, Department of Pediatric Dentistry, Erzurum, Turkey. Visualization, wrote original draft, reviewed and edited manuscript, contributed substantially to the conception and design of the study.

**Keywords:** anti-bacterial agents, endodontics, root canal disinfection.

## Abstract

**Purpose:**

Persistent bacterial contamination of the root canal system, particularly by *Enterococcus faecalis* (*E. faecalis*) and *Streptococcus mutans* (*S. mutans*), remains a major obstacle in endodontic therapy. Sodium hypochlorite (NaOCl) is widely recognized as the gold-standard irrigant due to its broad-spectrum antimicrobial properties. However, its cytotoxicity has prompted the exploration of alternative or adjunctive disinfection methods, including ozone therapy, diode lasers, and photodynamic therapy (PDT). This study aimed to evaluate and compare the antimicrobial efficacy of NaOCl, ozone, diode laser, and PDT—used alone or in combination with NaOCl—against *E. faecalis* and *S. mutans* in vitro.

**Materials and Methods:**

Standard strains (*E. faecalis* ATCC 29212 and *S. mutans* ATCC 25175) were inoculated into 96-well microplates and exposed to the designated treatments following standardized protocols. In the combination groups, NaOCl was applied first, followed immediately by the secondary modality without rinsing. Colony-forming units (CFUs) were quantified by plating on Brain Heart Infusion agar and incubating under appropriate conditions. Statistical significance was assessed using the Kruskal–Wallis test with post-hoc pairwise comparisons (p < 0.05).

**Results:**

NaOCl, both alone and in combination, completely eradicated both bacterial species. Among the alternative methods, ozone gas and PDT statistically significantly reduced *S. mutans* counts but were less effective against *E. faecalis*. Diode laser and ozonated water exhibited minimal antimicrobial activity. No synergistic enhancement was observed in the combination groups.

**Conclusions:**

NaOCl remains the most effective agent for root canal disinfection. Although ozone and PDT showed moderate efficacy—particularly against *S. mutans*—they may serve as adjunctive options when NaOCl use is limited. Further research is warranted to optimize these alternative approaches for clinical implementation.

The oral cavity hosts a wide array of pathogenic microorganisms.^3,22^ The complex anatomy of the root canal system enhances the pathogenic potential of these microbes, often leading to the development of inflammatory lesions in the periapical region.^2^ Among these pathogens, *Enterococcus faecalis *(*E. faecalis*) is recognized as the most persistent bacterial species responsible for recurrent endodontic infections. Its affinity for dentinal tubules, adaptability to diverse environmental conditions, resistance to antimicrobial agents, and genetic polymorphism make it particularly difficult to eradicate from root canals.^7^

While *Streptecoccus mutans* (*S. mutans*) is not frequently detected in root canals, it has been implicated as a potential initiator of pulpal infections.^2^ Although it is not typically the primary cause of persistent endodontic infections, its early colonization may play a key role in disease initiation. Therefore, the successful elimination of both *E. faecalis* and *S. mutans* is critical to achieving long-term success in endodontic therapy.^22^

Sodium hypochlorite (NaOCl) is widely regarded as the most commonly used irrigant in endodontic treatment due to its broad-spectrum antimicrobial activity, affordability, and ease of access.^16^ In addition to its effective dissolution of organic tissue, NaOCl also exhibits anti-inflammatory properties. However, it has several well-known limitations, including an unpleasant taste and odor, cytotoxic effects on periapical tissues and oral mucosa, and an inability to completely eliminate the smear layer.^1,10^ These limitations have prompted ongoing research into alternative or adjunctive disinfection strategies aimed at improving microbial control while minimizing adverse effects.

Ozone, diode lasers, and photodynamic therapy (PDT) are being increasingly explored as alternative or adjunctive methods in endodontic disinfection. Ozone is available in gas, aqueous, and oil forms and is frequently utilized in dental applications due to its strong oxidative potential, which significantly reduces pathogenic load within root canals.^9^ Diode lasers, typically operating at a wavelength of 810 nm in endodontic procedures, can penetrate dentinal tubules and effectively eradicate bacteria without promoting microbial resistance.^25^ PDT, a more recent and selective technique, involves the use of a non-toxic photosensitizer that, upon activation by low-level laser light, generates reactive oxygen species (ROS) in the presence of oxygen. These ROS induce oxidative damage to microbial cells, ultimately leading to their destruction.^21^ Each method offers unique antimicrobial advantages that may enhance clinical outcomes, especially in complex endodontic cases.

Numerous studies have evaluated the antimicrobial efficacy of various agents against *E. faecalis* and *S. mutans.* ^4,5,14,18,24^ However, to the best of our knowledge, no previous research has specifically investigated the antimicrobial performance of PDT against *S. mutans*. Moreover, comparative data directly assessing PDT alongside well-established agents such as NaOCl, ozone, and diode lasers remain scarce. This highlights an important gap in the literature and underscores the need for a more comprehensive assessment of PDT’s clinical potential.

In this context, the present study was designed to compare the antimicrobial efficacy of NaOCl, diode laser, ozone gas, and PDT, both individually and in combination with NaOCl, against *E. faecalis* and *S. mutans*. The null hypothesis tested was that there would be no statistically significant differences in antimicrobial efficacy among the evaluated agents and their combinations.

## MATERIALS AND METHODS

### Ethical Approval 

The protocol for our study was approved by the Ataturk University Faculty of Medicine’s Local Ethics Committee (1/35, 31 January 2025).

### Bacterial Strains and Culture Conditions

This in-vitro study utilized standard strains of *E. faecalis* (ATCC 29212) and *S. mutans* (ATCC 25175). Until use, bacterial stocks were stored at -80°C in the microbiology laboratory of Ataturk University, Faculty of Pharmacy. For experimental preparation, bacteria were subcultured on Brain Heart Infusion (BHI) agar (Difco; Detroit, MI, USA). *S. mutans* was incubated overnight at 37°C in a microaerophilic environment (95% air and 5% CO₂), while *E. faecalis* was incubated under aerobic conditions. From these fresh cultures, suspensions were prepared in BHI broth and adjusted to a turbidity of 0.5 McFarland standard (1 × 10^8^ CFU/ml).

### Preparation of Experimental Wells

The antimicrobial activity tests were conducted using sterile 96-well microplates, designed to simulate clinical root canal contamination. Two separate microplates were used—one for *E. faecalis* and one for *S. mutans*. For each treatment group, 9 wells were assigned per bacterial species.

A 50-µl aliquot of the bacterial suspension was added to each well and incubated at 37°C for 1 h to allow bacterial adherence. After incubation, wells were aspirated to remove the medium, and the plates were left to dry under sterile conditions.

### Antimicrobial Treatments

The wells were randomly assigned to one of the following treatment groups:

NaOCl
Diode laser
PDT
Ozone gas
Ozonated water
NaOCl + diode laser
NaOCl + PDT
NaOCl + ozone gas
NaOCl + ozonated water
Control (no treatment)

All interventions were performed according to standardized protocols. For the combination groups, NaOCl was applied directly into the wells containing the bacterial suspension and culture medium. Without aspirating or rinsing the contents, the corresponding secondary treatment (diode laser, PDT, ozone gas, or ozonated water) was applied immediately thereafter. Control wells did not receive any treatment.

A 2.5% NaOCl solution was prepared and directly applied to 200-µl wells containing bacterial suspensions. The solution was transferred using sterile, single-use 5-ml dental syringes and allowed to remain in direct contact with the bacteria for 60 s without disturbance to ensure effective antimicrobial interaction.

Ozone gas was generated using the Ozonytron XP device (Munich, Germany) and applied for 1 min at 60% concentration, while ozonated water was prepared by saturating sterile NaCl solution with 80% of the generated ozone gas. The prepared solution was similarly transferred to 200-µl wells containing bacterial suspensions using sterile, disposable 5-ml dental syringes and similarly allowed to remain in direct contact with the bacteria for 60 s without disturbance.

For the PDT (EasyinSmile; Changsha City, China) group, toluidine blue was used as the photosensitizing agent. Following staining of the bacteria with the photosensitizer for 1 min, red light at a wavelength of 630 nm was applied from a distance of approximately 0.5 cm. The irradiation was performed for 30 s, following the manufacturer’s recommended parameters for endodontic applications.

The diode laser (Doctor Smile; Brendola, Italy) was applied at a wavelength of 808 nm, with a power output of 6 W, in canal-sterilization mode. Laser irradiation was performed from a distance of approximately 0.5 cm for 10 s on the wells containing bacterial suspensions.

Following the application of all agents, no irrigation was performed, and the residual bacterial counts in the wells were measured immediately after the procedures.

### Colony Counting Procedure

Following treatment, 100 µl of sterile physiological saline was added to each well and gently agitated for 1 min. Then, 50 µl of the solution was aspirated and plated onto BHI agar. Plates inoculated with *S. mutans* were incubated at 37°C in 95% air and 5% CO_2_, while those with *E. faecalis* were incubated under aerobic conditions. After overnight incubation, bacterial growth was evaluated by counting colony-forming units (CFUs). All procedures were performed in triplicate for each group, and all colony counts were conducted by a blinded evaluator to ensure consistency and eliminate bias.

### Statistical Analysis

Normality was assessed using the Shapiro-Wilk test, and the homogeneity of variances was tested using Levene’s test. Since the assumptions for parametric analysis were not met, non-parametric tests were used. The Kruskal–Wallis test was applied to evaluate overall differences among groups, followed by post-hoc pairwise comparisons. A p-value < 0.05 was considered statistically significant. Statistical analyses were performed using SPSS software (version 22.0, IBM; Armonk, NY, USA).

## RESULTS

**Fig 1:**
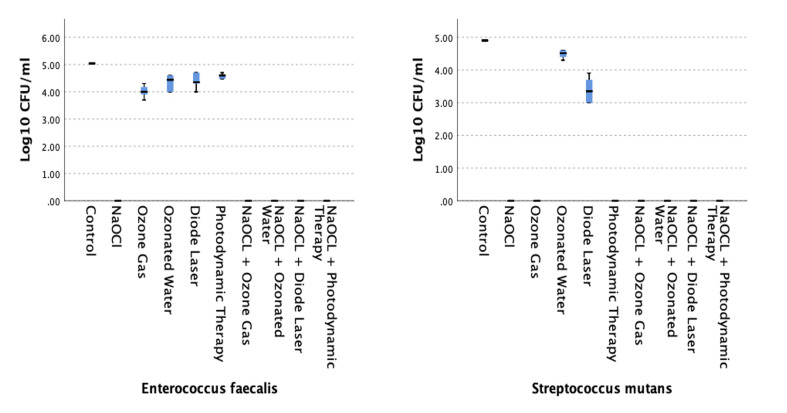
Antibacterial effects of tested treatments against (a) *Enterococcus faecalis* and (b) *Streptococcus mutans*. NaOCl and its combinations resulted in complete bacterial elimination (100% reduction), while alternative treatments showed variable efficacy depending on the bacterial species.

The antimicrobial activities of all tested agents and their combinations were evaluated against *E. faecalis* and *S. mutans*, as illustrated in Fig 1 and detailed in Tables 1 and 2.

**Table 1:** Pairwise comparisons of bacterial colony counts for *Enterococcus faecalis* between experimental groups based on the Kruskal–Wallis test followed by post-hoc pairwise comparisons

	Control	Diode laser	NaOCl + diode laser	NaOCl+ ozonated water	NaOCl + ozone gas	NaOCl + photodynamic therapy	NaOCl	Ozonated water	Ozone gas
Control									
Diode laser	0.149								
NaOCl + diode laser	<0.001*	0.003*							
NaOCl + ozonated water	<0.001*	0.003*	1						
NaOCl + ozone gas	<0.001*	0.003*	1	1					
NaOCl + photodynamic therapy	<0.001*	0.003*	1	1	1				
NaOCl	<0.001*	0.003*	1	1	1	1			
Ozonated water	0.113	0.887	0.004*	0.004*	0.004*	0.004*	0.004*		
Ozone gas	0.018*	0.353	0.037*	0.037*	0.037*	0.037*	0.037*	0.431	
Photodynamic therapy	0.33	0.639	<0.001*	<0.001*	<0.001*	<0.001*	<0.001*	0.542	0.162
*Statistically significant difference between groups (p < 0.05).

**Table 2:** Pairwise comparisons of bacterial colony counts for *Streptococcus mutans* between experimental groups based on the Kruskal–Wallis test followed by post-hoc pairwise comparisons

	Control	Diode laser	NaOCl + diode laser	NaOCl+ ozonated water	NaOCl + ozone gas	NaOCl + photodynamic therapy	NaOCl	Ozonated water	Ozone gas
Control									
Diode laser	0.142								
NaOCl+ diode laser	<0.001*	0.003*							
NaOCl + ozonated water	<0.001*	0.003*	1						
NaOCl + ozone gas	<0.001*	0.003*	1	1					
NaOCl + photodynamic therapy	<0.001*	0.003*	1	1	1				
NaOCl	<0.001*	0.003*	1	1	1	1			
Ozonated water	0.463	0.463	<0.001*	<0.001*	<0.001*	<0.001*	<0.001*		
Ozone gas	<0.001*	0.003*	1	1	1	1	1	<0.001*	
Photodynamic therapy	<0.001*	0.003*	1	1	1	1	1	<0.001*	1
*Statistically significant difference between groups (p < 0.05).

NaOCl, both as a stand-alone agent and in combination with diode laser, PDT, ozone gas, or ozonated water, demonstrated complete antibacterial activity, resulting in a 100% reduction in colony-forming units (CFUs) for both *E. faecalis *and* S. mutans*. These groups showed no detectable bacterial growth post-treatment, confirming the superior efficacy of NaOCl across all tested conditions.

On the other hand, diode laser, ozone gas, ozonated water, and PDT applied individually produced variable levels of antimicrobial effect. Although these treatments reduced bacterial counts to varying degrees, not all comparisons to the control group reached statistical significance. Specifically, for *E. faecalis*, the diode laser (p = 0.149), ozonated water (p = 0.113), and PDT (p = 0.33) groups did not differ statistically significantly from the control. Only ozone gas treatment resulted in a statistically significant reduction (p = 0.018).

In *S. mutans*, ozone gas (p < 0.001) and PDT (p < 0.001) showed statistically significant reductions in CFUs compared to control, while diode laser (p = 0.142) and ozonated water (p = 0.463) did not.

Statistical analyses using the Kruskal–Wallis test followed by post-hoc pairwise comparisons consistently showed that NaOCl-containing groups were statistically significantly more effective than all other treatments, including the alternative methods, for both bacterial species (p < 0.001).

Among the alternative (non-NaOCl) treatments, ozone gas showed the highest overall antimicrobial activity, achieving statistically significant reductions in both bacterial species compared to the control group. PDT was similarly effective against *S. mutans* but failed to produce statistically significant reductions in *E. faecalis*. Diode laser and ozonated water were the least effective treatments, showing no statistically significant reduction in either bacterial species when compared to the control. These findings suggest that, although inferior to NaOCl, ozone gas and PDT may offer some clinical benefit, particularly against *S. mutans*.

## DISCUSSION

Effective disinfection of the root canal system is a critical prerequisite for the long-term success of endodontic treatment. However, the complex anatomy of root canals often allows persistent colonization by resistant microorganisms such as *E. faecalis* and, to a lesser extent, *S. mutans*. These species are associated with secondary infections and treatment failure.^20,26^ Therefore, identifying and comparing the efficacy of different antimicrobial strategies remains a key focus in endodontic research.

Numerous studies have confirmed the potent antimicrobial properties of NaOCl, establishing it as the gold standard irrigant in endodontics.^6,16^ Its effectiveness stems from its broad-spectrum antimicrobial action and its ability to dissolve organic tissues, which makes it especially valuable in eliminating biofilm within the root canal system. Nevertheless, NaOCl has notable drawbacks, including cytotoxicity to periapical tissues and its inability to completely remove the smear layer, which have driven interest in alternative or adjunctive methods.^12,23^ Among these, ozone gas, diode lasers, and PDT have gained attention as promising supplementary techniques. Ozone’s potent oxidative properties underlie its microbial inactivation capacity; diode lasers offer the advantage of deep penetration into dentinal tubules; and PDT, a more recent development, selectively targets bacterial cells via a photosensitizer activated by low-level laser light.^11,15,17^

This study was designed to evaluate and compare the antimicrobial efficacy of NaOCl, ozone, diode lasers, and PDT, both individually and in combination, against *E. faecalis* and *S. mutans*. While *E. faecalis* has been extensively studied in the endodontic literature, data on *S. mutans* remain limited. Although *S. mutans* is not typically associated with persistent infections, it is known to play a key role in the initiation of endodontic pathology and has been isolated from root canals in various clinical settings.^13^ Notably, the assessment of PDT’s antimicrobial performance against *S. mutans* in this context represents a novel contribution. Furthermore, the scarcity of studies that directly compare PDT with other established methods such as NaOCl, ozone, and diode lasers underscores the relevance and originality of this research. The findings of this study may serve to guide the selection and optimization of antimicrobial protocols in clinical endodontic practice.

The present findings revealed that all NaOCl-containing groups led to complete elimination of both *E. faecalis* and *S. mutans*, achieving 100% reduction in viable bacterial counts. This outcome is consistent with previous research highlighting NaOCl’s robust antimicrobial properties and reinforces its status as the most effective agent for root canal disinfection.^19^ Notably, combining NaOCl with other modalities such as ozone, PDT, or diode lasers did not confer any additional antimicrobial benefit. This may be attributable to the fact that NaOCl, applied directly to wells containing both bacterial suspension and culture medium, remained present during the subsequent application of the secondary agent. Thus, its dominant antimicrobial effect likely masked or overshadowed any potential additive or synergistic contributions from the adjunctive treatments.

In contrast to the NaOCl-containing groups, diode laser, ozone, and PDT applied individually resulted in only partial suppression of bacterial growth. The antimicrobial performance of these modalities varied by bacterial species, demonstrating greater effectiveness against *S. mutans* than *E. faecalis*. This disparity is likely due to the inherent resistance mechanisms of *E. faecalis*, including its capacity to invade dentinal tubules, persist in nutrient-limited environments, and resist oxidative and chemical stressors.^11,17^ These characteristics make *E. faecalis* particularly difficult to eliminate and may necessitate more aggressive or combinatory treatment approaches in clinical practice.

Although PDT represents a promising antimicrobial approach, its limited performance against resistant organisms such as *E. faecalis* underscores the need for further optimization. Enhancements may include the use of more potent or targeted photosensitizers, refinement of laser energy parameters, and extended exposure durations.^8^ Likewise, the efficacy of physical disinfection methods such as ozone gas and diode lasers may be improved through recalibration of key variables including application time, power settings, and repetition protocols, as emphasized in previous studies.^11,15^

Although the degree of antimicrobial activity varied among the tested agents, all treatment groups demonstrated statistically significant reductions in bacterial counts when compared to the untreated control. These findings suggest that even the less effective methods may have clinical utility as adjuncts, particularly in scenarios where the use of NaOCl is contraindicated or limited, such as in pediatric endodontics. Based on the statistical outcomes, the null hypothesis—stating that there would be no statistically significant difference in antimicrobial efficacy among the agents and their combinations—was therefore rejected.

Several limitations should be acknowledged in interpreting these findings. First, as this study was conducted under in-vitro conditions, it lacks the biological complexity of the in-vivo root canal environment, including dynamic fluid exchange, host immune interactions, and tissue buffering capacity. These factors may influence the antimicrobial efficacy of irrigants in clinical applications. Second, bacterial viability was assessed using conventional colony-forming unit counts, which do not account for viable but non-culturable organisms. Therefore, the actual bacterial survival rate may have been underestimated. Incorporating molecular techniques such as quantitative PCR (qPCR) in future studies could provide a more comprehensive evaluation of microbial persistence, particularly in assessing biofilm-associated and non-culturable bacterial populations.

## CONCLUSION

NaOCl was found to be the most effective agent for root canal disinfection, completely eliminating *E. faecalis* and *S. mutans*. Although diode laser, ozone, and photodynamic therapy demonstrated only partial antimicrobial activity—particularly against *S. mutans*—they still achieved statistically significant reductions compared to the control. These findings highlight the potential of such alternative methods as adjuncts in endodontic therapy, especially when NaOCl use is contraindicated or limited. Future studies should focus on refining these methods and investigating their possible synergistic effects in more clinically relevant models.

## References

[ref1] Akbulut MB, Unverdi Eldeniz A (2019). In vitro antimicrobial activity of different electrochemically-activated solutions on enterococcus faecalis. Eur Oral Res.

[ref2] Atila-Pektas B, Yurdakul P, Gulmez D, Gorduysus O (2013). Antimicrobial effects of root canal medicaments against enterococcus faecalis and streptococcus mutans. Int Endod J.

[ref3] Bardakcı E, Yıldız S, Yazmacı B, Doğan ME, Mumcu K, Doğan MS (2025). Evaluation of pediatric patients’ general health status prior to dental treatment under general anesthesia: A retrospective study. Children-Basel.

[ref4] Celikel P, Ceyhan B, Buyuksefil M, Levchenko A, Sengul F, Simsek Derelioglu S (2025). Antimicrobial activity of different irrigation solutions on enterococcus faecalis in the root canal of the primary teeth-an in vitro comparative study. JOCPD.

[ref5] Dai S, Xiao G, Dong N, Liu F, He S, Guo Q (2018). Bactericidal effect of a diode laser on in human primary teeth-an in vitro study. BMC Oral Health.

[ref6] del Carpio-Perochena A, Bramante CM, de Andrade FB, Maliza AG, Cavenago BC, Marciano MA (2015). Antibacterial and dissolution ability of sodium hypochlorite in different phs on multi-species biofilms. Clin Oral Investig.

[ref7] Deng DM, Hoogenkamp MA, Exterkate RA, Jiang LM, van der Sluis LW, Ten Cate JM (2009). Influence of Streptococcus mutans on Enterococcus faecalis biofilm formation. J Endod.

[ref8] Di Taranto, V, Libonati A, Montemurro E, Gallusi G, Campanella V (2022). Antimicrobial effects of photodynamic and high-power laser endodontic therapy on patients with necrotic pulp and periapical lesion. J Biol Regul Homeost Agents.

[ref10] Gamal-AbdelNaser A, Elnaggar A, Mekawy M, Boshra G, Ghareeb N (2025). Sodium hypochlorite accident-complications, management and potential prevention: A report of three cases. Front Oral Maxillofac Med.

[ref11] Gutknecht N, Franzen R, Schippers M, Lampert F (2004). Bactericidal effect of a 980-nm diode laser in the root canal wall dentin of bovine teeth. J Clin Laser Med Surg.

[ref12] Haapasalo M, Endal U, Zandi H, Coil MJ (2005). Eradication of endodontic infection by instrumentation and irrigation solutions. Endodontic topics,.

[ref13] Lima AR, Herrera DR, Francisco PA, Pereira AC, Lemos J, Abranches J (2021). Detection of in symptomatic and asymptomatic infected root canals. Clin Oral Investig.

[ref14] Malik S, Mulla M, Saheb SAK, Abdulaziz Alessa N, Chougule VT, Mulla M (2024). Evaluation of antimicrobial effect of ginger, apple cider vinegar against. J Pharm Bioallied Sci.

[ref15] Meire MA, De Prijck K, Coenye T, Nelis HJ, De Moor RJ (2009). Effectiveness of different laser systems to kill in aqueous suspension and in an infected tooth model. Int Endod J.

[ref16] Mutluay AT, Mutluay M (2015). Sodıum hypochlorıte in endodontıcs. J Dent Fac Ataturk Univ.

[ref17] Nagayoshi M, Kitamura C, Fukuizumi T, Nishihara T, Terashita M (2004). Antimicrobial effect of ozonated water on bacteria invading dentinal tubules. J Endod,.

[ref18] Novozhilova N, Babina K, Polyakova M, Sokhova I, Sherstneva V, Zaytsev A (2024). The effect of different compositions and concentrations of etidronate-containing irrigants on the antibacterial activity of sodium hypochlorite against enterococcus faecalis and candida albicans. J Dent.

[ref19] Orozco-Gallego MJ, Pineda-Vélez EL, Rojas-Gutiérrez WJ, Rincón-Rodríguez ML, Agudelo-Suárez AA (2025). Effectiveness of irrigation protocols in endodontic therapy: An umbrella review. J Dent.

[ref20] Parga A, Mattu J, Belibasakis NG, Kline KA, Leprince J, Manoil D (2025). A polymicrobial perspective into the ecological role of in dental root canal infections. NPJ Biofilms Microbiomes.

[ref21] Plotino G, Grande NM, Mercade M (2019). Photodynamic therapy in endodontics. Int Endod J.

[ref22] Sharma R, Reddy VK, Prashant G, Ojha V, Kumar NP (2014). Antimicrobial and anti-adherence activity of various combinations of coffee-chicory solutions on streptococcus mutans: An in-vitro study. J Oral Maxillofac Pathol.

[ref23] Siqueira JF Rôças IN (2008). Clinical implications and microbiology of bacterial persistence after treatment procedures. J Endod.

[ref24] Tognetti VM, Toledo EDS, Alves TM, Rizzardi KF, Parisotto TM, Pascon FM (2024). Effect of two irrigating solutions on antimicrobial activity and clinical and radiographic success after endodontic treatment in primary teeth: A randomized clinical trial. Clin Oral Investig.

[ref25] Vinothkumar TS, Renugalakshmi A, El-shamy FMM, Homeida HE, Hommedi AI M, Safhi MYA (2020). Antibacterial effect of diode laser on different cariogenic bacteria: An -study. Niger J Clin Pract.

[ref26] Wu B, Zhou Z, Hong X, Xu Z, Xu Y, He Y (2025). Novel approaches on root canal disinfection methods against. J Oral Microbiol.

